# Traumatic Isolated Trapezium Dislocation without Fracture: A Case Report and Review of the Literature

**DOI:** 10.1155/2016/1798941

**Published:** 2016-03-23

**Authors:** Robert M. Kenyon, Enda G. Kelly, Benny Padinjarathala

**Affiliations:** Trauma and Orthopaedics Department, University Hospital Waterford, Waterford, Ireland

## Abstract

Isolated dislocation of the trapezium is an uncommon injury. There are sixteen cases to date reported in the literature. The management of these cases has varied from complete excision to open or closed reduction, with or without percutaneous wiring. This paper presents a case of an isolated dislocation of the trapezium without fracture, managed with closed reduction and percutaneous wiring, resulting in a good functional outcome.

## 1. Introduction

Traumatic dislocation of the trapezium is a relatively rare injury [1]. The most common mechanism associated with this injury tends to be either a direct blow or a crush injury [2]. Dislocation of the trapezium without an associated carpal or metacarpal fracture is rarer still [3–6]. The strong dorsal ligamentous attachments to the trapezium ensure that traumatic force will tend to result in first metacarpal fractures in preference to trapezium dislocation [1]. Given the propensity for a grinding or crushing force in these injuries and the likelihood of extensive associated soft tissue damage, these cases often present as open injuries. We describe the case of a closed trapezium dislocation with no associated fracture.

## 2. Case Presentation

A 47-year-old right hand dominant male working as a stable hand presented to the emergency department of a tertiary trauma and orthopedic referral center following a crush injury to the left hand. The injury had occurred while the patient had been assisting a friend to park a 4 × 4 vehicle. The vehicle had been reversing slowly towards a wall with the patient's hand behind the vehicle. The mechanism of injury suggested axial loading of the thumb between the vehicle and the wall. He presented directly to the emergency department with severe pain, swelling, and an obvious deformity at the wrist. The patient had no background medical or surgical history of note and reported no previous injuries to the affected limb. He was an active smoker of 30 cigarettes per day.

On examination in the emergency department, he had significant pain and swelling in the left wrist and forearm. There was a superficial abrasion over the dorsal aspect of the affected hand; however, this was considered to be a closed injury. There was significant tenderness over the carpals, particularly at the trapezium, with associated reduced range of motion at the wrist and thumb. There was no neurovascular compromise or associated tendon injury.

**Figure 1:**
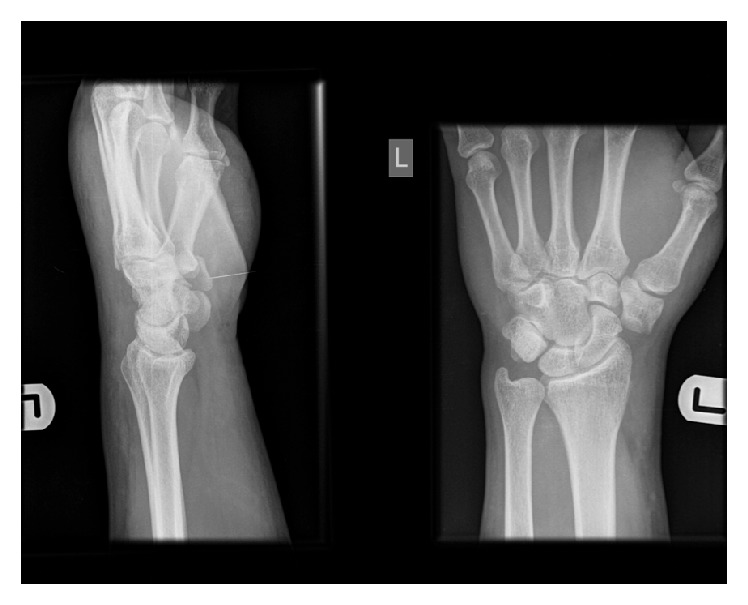
Preoperative radiographs.

The AP radiograph reveals disruption of the continuation of arc I between the proximal border of the triquetrum and the proximal border of the trapezium, with the trapezium displaced in a radial direction (Figure 1). This represents a volar dislocation of the trapezium. There was no associated fracture of the trapezium or any other associated bony injuries.

**Figure 2:**
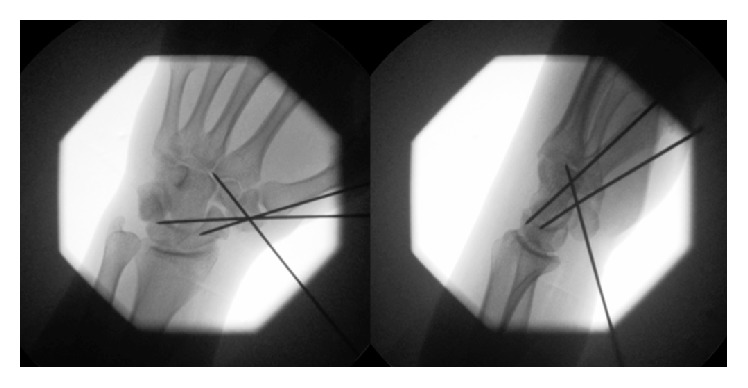
Intraoperative radiographs.

The patient subsequently underwent closed manipulation under general anesthesia. The surgeon achieved anatomical reduction by gripping the patient's thumb in one hand and applying a distracting force, while simultaneously applying pressure on the radial aspect of the trapezium with the thumb of the surgeon's other hand. The trapezium was stabilized with three 1.6 mm Kirschner wires percutaneously inserted from the radial side. One wire passed from trapezium to scaphoid, one wire passed from trapezium to capitate, and one wire passed from trapezium to trapezoid (Figure 2). The wrist was immobilized in a below-elbow molded POP thumb spica cast. The patient was discharged later on the same day.

**Figure 3:**
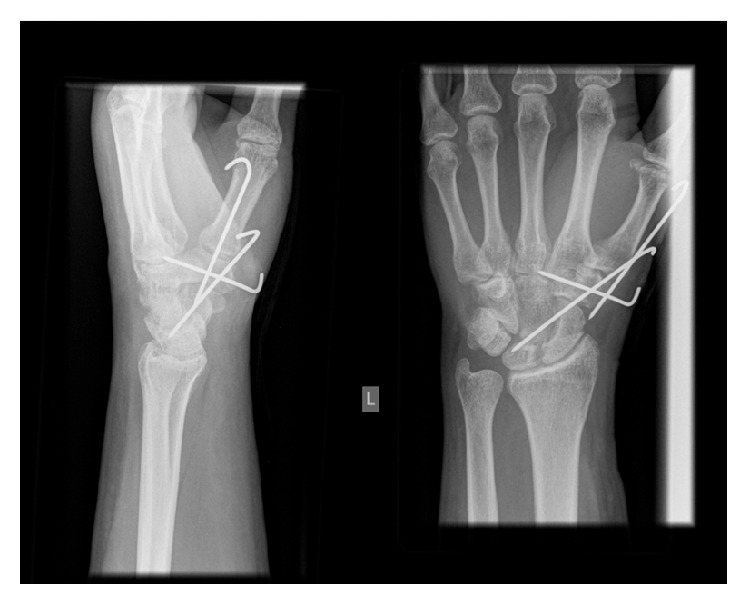
Radiographs at 6 weeks.

**Figure 4:**
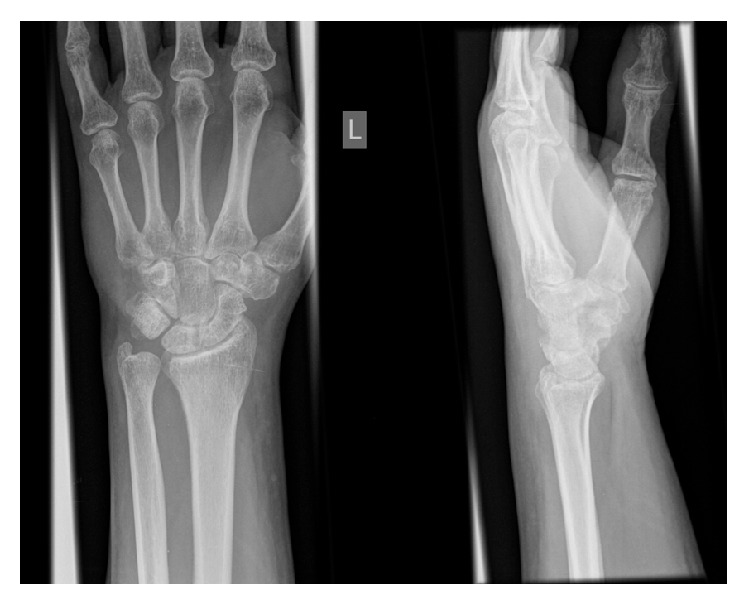
Radiographs at 3 months.

The patient remained in cast for a total of six weeks at which point the percutaneous wires were removed in the outpatient department and the hand and wrist were mobilized (Figure 3). He was referred to the local hand therapy service. Follow-up examination at three months revealed excellent range of motion (wrist flexion 60°, wrist extension 30°, pronation 80°, supination 80°, and radial abduction at base of thumb 60°). He had returned to full manual work activities at 8 weeks postoperatively and was no longer requiring analgesia (Figure 4).

## 3. Discussion

A systematic method for evaluating the alignment of the carpal bones is essential at the initial assessment of wrist injuries to ensure that uncommon fracture/dislocations are diagnosed and treated early. The assessment of carpal alignment in trauma was first described in detail by Gilula in 1979 [7]. There are three arcs within the wrist seen on anteroposterior plain film radiograph, which if disrupted strongly suggest an abnormality of alignment at the site of disruption. Arc I is the convex curve along the proximal surfaces of scaphoid, lunate, and triquetrum, arc II is the concave curve along the distal surfaces of scaphoid, lunate, and triquetrum, and arc III is the convex curve along the proximal surfaces of capitate and hamate. Carpal dislocations generally occur as a result of high-energy trauma. Dislocations of the trapezium tend to occur in either a volar or a radiodorsal direction [2]. The relative strength of the ligaments attached to the trapezium dorsally compared to those attaching to the volar surface results in a greater propensity for volar dislocation [8].

There are only thirteen existing reports depicting a total of sixteen cases of isolated trapezium dislocations published in the literature since the 1950s [1, 3–6, 8–15]. These include both open and closed dislocations and the majority of these involve an associated fracture of either the trapezium or the surrounding bones. Five cases report an isolated trapezium dislocation without any associated fracture of the trapezium or adjacent bones [3–6]. Of these, two report an open injury [4] and three report a closed injury [3, 5, 6]. In addition, there were also four reported cases that involved only minor avulsion type fractures of the trapezium [1, 8, 9, 12].

Excision of the trapezium has been described in the treatment of isolated dislocation in which reduction was not possible [4, 8]. The potential risk of avascular necrosis has been suggested as the rationale for such an approach [4]. However, in general, this option is considered as a salvage technique only [2]. In the majority of reported cases, percutaneous fixation with Kirschner wires has been used with acceptable results [1, 5, 9–12, 14, 15]. An open reduction technique has been employed in all cases where the fracture was open. The management of closed dislocations has varied. Some studies advocate an open reduction, regardless of whether the injury is open or closed to begin with [9]. Three cases demonstrated a successful closed reduction [3, 6, 11] and in the case of McKie et al. and Vente and de Ruiter these dislocations were successfully treated without the use of percutaneous wiring [3, 6].

## 4. Conclusion

This case demonstrates that a closed, isolated dislocation of the trapezium with no associated fracture can be safely and effectively treated with closed reduction and percutaneous wire fixation. The following learning points can be gleaned from the case and supporting literature:

- A systematic method of evaluating carpal alignment is essential to ensure early pick-up of uncommon carpal injuries.
- Axial loading of the wrist may result in an isolated dislocation of the trapezium without associated fractures.
- Closed reduction should be attempted with percutaneous wire fixation.
- Open reduction should be employed if there is a failure to achieve adequate reduction.
- Excision of the trapezium is rarely necessary.
